# Compensation for Process and Temperature Dependency in a CMOS Image Sensor

**DOI:** 10.3390/s19040870

**Published:** 2019-02-19

**Authors:** Shuang Xie, Albert Theuwissen

**Affiliations:** 1 EI Lab, Delft University of Technology, 2628 CD Delft, The Netherlands; 2 Harvest Imaging, 3960 Bree, Belgium; albert@harvestimaging.com

**Keywords:** temperature sensors, delta-sigma (Δ-σ) modulator, CMOS image sensor (CIS), thermal compensation, dark current, dark signal non-uniformity (DSNU), process variability, process variations, conversion gain (CG)

## Abstract

This paper analyzes and compensates for process and temperature dependency among a (Complementary Metal Oxide Semiconductor) CMOS image sensor (CIS) array. Both the analysis and compensation are supported with experimental results on the CIS’s dark current, dark signal non-uniformity (DSNU), and conversion gain (CG). To model and to compensate for process variations, process sensors based on pixel source follower (SF)’s transconductance *g_m_*_,*SF*_ have been proposed to model and to be compared against the measurement results of SF gain *A_SF_*. In addition, *A_SF_*’s thermal dependency has been analyzed in detail. To provide thermal information required for temperature compensation, six scattered bipolar junction transistor (BJT)-based temperature sensors replace six image pixels inside the array. They are measured to have an untrimmed inaccuracy within ±0.5 °C. Dark signal and CG’s thermal dependencies are compensated using the on-chip temperature sensors by at least 79% and 87%, respectively.

## 1. Introduction

The pixels in a CMOS image sensor (CIS) array, as all semiconductor devices, are sensitive to process, voltage, and temperature (PVT) variations, which give rise to (Fixed Pattern Noise) FPN, including dark current or degraded conversion gain (CG) with temperature [1]. Voltage supply dependency and mismatches among source follower (SF)’s threshold voltages (*V_TH_*) can be eliminated by correlated double sampling (CDS), a state-of-the art technique in CIS [1]. However, CDS cancels the offset rather than the gain mismatches. Earlier efforts to further suppress FPN’s gain mismatch have been reported in [2], which adjusts both the gain and the offset in each digital pixel containing a 10 bit analog-to-digital converter (ADC). However, its 50 µm pixel pitch excludes its general usage in CIS and it does not consider any thermal effect on dark current or CG. To sense temperature inside a pixel array, previous publications [3,4] integrate seamless temperature sensors inside a CIS array despite no dark compensation being performed. Meanwhile, it is reported in [5] that the CG decreases with temperature, although the virtual mechanisms that contribute to the temperature dependency of pixel SF are not clearly defined. Besides, the work in [5] is incapable of compensating on-the-fly for linearity degradation with dynamic temperature change, not only because it has no temperature sensor on-chip, but also due to its linearity compensation method. In addition, [5] requires an accurate input light or voltage source for its algorithm. Reference [6] discusses the thermal dependence of Pinned Photodiode (PPD). In contrast to the aforementioned publications, this paper explores the possibilities to predict and compensate for process and temperature dependency without requiring any accurate input voltage or light source, in the following aspects: (1) Process sensors based on pixel SF’s transconductance *g_m_*_,*SF*_ have been analyzed and measured, using column current biasing circuits with dynamic element matching (DEM). (2) SF’s voltage gain *A_SF_* is modeled based on the measured *g_m_*_,*SF*_ from the process sensors in (1) and compared against its measurement results. Particularly, the mechanisms that contribute to the thermal and process dependency of *A_SF_* have been fully analyzed and quantized, supported with its measurement results and extractions, using constant current and constant *g_m_* biasing, respectively. (3) The CIS’s CG and dark current’s temperature dependency are measured and dynamically compensated using in-pixel temperature sensors. Using the six temperature sensors which are measured to have untrimmed inaccuracy within ±0.5 °C, the average dark signal’s temperature dependency is compensated by at least 79%, and the CG by 87%. In general, this paper proposes self-calibrating the CIS using SF’s own process element–*g_m_*_,*SF*_, when employing DEM, thus eliminating any need for an accurate external voltage or light source. In other words, each of the image pixel’s SF serves as a process sensor. The column readout circuits are 14 bit 1st-order delta-sigma ADCs (DSADC), which are less sensitive to process and temperature variations, due to their feedback loop, equaling their digital bit stream (bs) outputs to the analog inputs. Both the resolution and noise of the DSADC are less than 10 µV.

This paper is organized as follows. Section 2 discusses the theoretical thermal and process dependency of pixel SF’s gain *A_SF_* and transconductance *g_m_*_,*SF*_, along with the process and the temperature sensors. Section 3 shows the measurement results of the temperature and the process sensors, and models the SF’s *A_SF_* using the process sensors’ measured *g_m_*_,*SF*_. The modeled *A_SF_* is compared against *A_SF_*’s measurement results, in relation to temperature and process variations. Section 4 shows the measurement results of CG and dark signal non-uniformity (DSNU), along with their thermally compensated results using the proposed in-pixel temperature and process sensors. Section 5 concludes this paper.

## 2. Pixel SF’s Temperature and Process Dependency, Process Sensor, and Temperature Sensor

### 2.1. Pixel SF’s Temperature and Process Dependency

**Figure 1:**
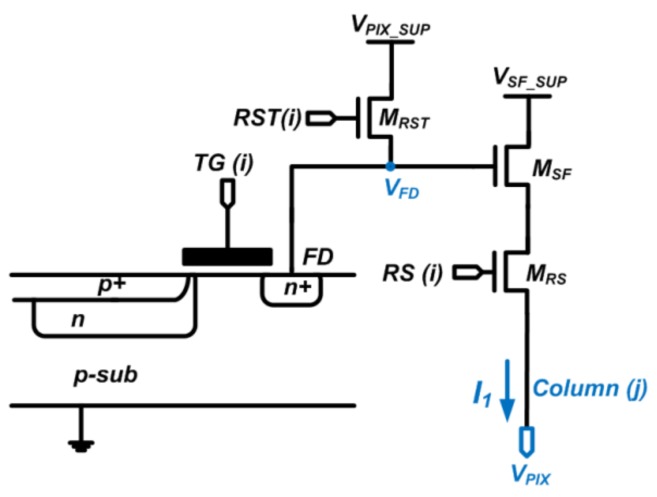
Schematic of four-transistor pinned-photodiode (4T PPD) image pixel based process sensor.

Figure 1 shows a four-transistor pinned-photodiode (4T PPD) CIS pixel. In most CIS technologies, a pixel SF is different from its alternative outside the array, as each employs different mask layers from the other. For this reason, the product design kits (PDK) intended for SF outside the array were unsuitable to simulate the in-pixel SF. This statement will be supported with the measurement results of SF’s *V_TH_*, which were much lower than the normal value of around 700 mV, as will be shown in Section 3. The analysis in this section includes two types of biasing circuits for pixel SF: constant current and constant *g_m_* biasing.

A pixel SF’s gain *A_SF_* can be expressed as [7]

(1) $$A_{SF}\;=\;\frac{g_{m,SF}}{\left(g_{m,SF}{+g}_{mb,SF}\right)+\frac{1}{R_{L}}},$$

where

(2) $$g_{mb,SF}\;=\;\frac{\gamma \cdot g_{m,SF}}{2\sqrt{{2\Phi }_{F}{+V}_{SB}}},$$

while source-body voltage *V_SB_* = *V_PIX_* if neglecting the voltage drop on the Row Select (RS) switch, and *V_PIX_* is positively correlated with the level of *g_m_*_,*SF*_ [5]. *R_L_* is the output impedance of the current source that provides *I*_1_ in Figure 1. *γ* is often called the body-effect constant, and *Φ_F_* is the Fermi potential of the body. *g_mb_*_,*SF*_ is the transconductance associated with body effect. If neglecting 1*/R_L_* in Equation (1), *A_SF_* decreases as *g_m_*_,*SF*_ falls, as *g_mb_*_,*SF*_ decreases in a much slower rate than that of *g_m_*_,*SF*_ due to the existence of *Φ_F_*. The CG of a CIS can be expressed as

(3) $${CG}_{CIS}{\;=\;CG}_{FD}\cdot A_{SF},$$

where *CG_FD_ = q/C_FD_*, where *C_FD_* is the total floating diffusing capacitance. Thus, the temperature and process dependency of *A_SF_* can convert into that of the total conversion gain *CG_CIS_*. Its process variations can cause FPN, such as DSNU. *g_m_*_,*SF*_ *=* *√* (2*µ_n_C_ox_W/L*·*I*) where *µ_n_* is the surface carrier mobility, *C_ox_* is the gate capacitance per unit area, *W, L*, and *I* are the width, length, and current of the SF, respectively. If the current *I* is designed to be constant, *g_m_*_,*SF*_ decreases with temperature, as the thermal coefficient of the surface carrier mobility *µ_n_ = µ_0_*(*T/T*_0_)^−α^ where *α* is usually from 1.5 to 3 [8] and *µ*_0_ is its value at absolute zero temperature. As a result, *A_SF_* decreases with temperature as well. Therefore, if the *A_SF_* can be predicted based on a simple measurement of the SF alone, it would help to define the *CG_CIS_’s* temperature coefficient as well as its process variations in each pixel. For this reason, we propose a process sensor based on (i) the measurement of *g_m_*_,*SF*_ and (ii) circuit simulation results of 1/*R_L_*. *R_L_* is located outside the pixel array so has its PDK model, unlike those transistors inside the image pixel. Then, *A_SF_* can be figured out using the aforementioned steps (i) and (ii).

### 2.2. Process Sensors

**Figure 2:**
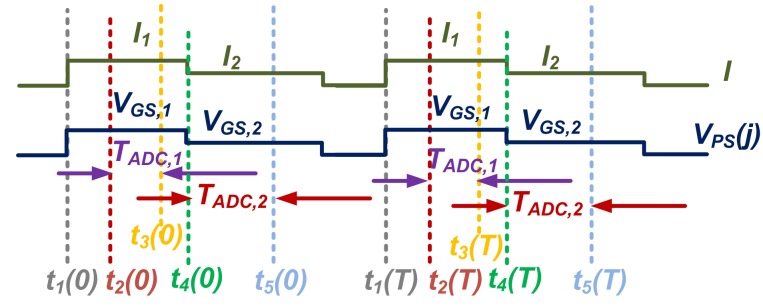
Proposed process sensor’s timing diagram.

To calibrate *A_SF_* as mentioned in Section 2.1, the proposed process sensor was based on the pixel SF itself, as shown Figure 1. Its timing diagram is shown in Figure 2. Between *t*_1_(0) to *t*_1_(*T*) is one conversion cycle; the same applies to *t*_2_(0) to *t*_2_(*T*), etc. During *T_ADC_*_,1_ and *T_ADC,2_*, the output voltage *V_PIX_* that corresponds to *V_GS_*_,1_ and *V_GS_*_,2_, was quantized, sequentially. The row reset *RST* has to be on and its voltage level has to meet the condition of: *V_RST_ > V_PIX_ + V_TH_* (*V_TH_* is the threshold voltage of *M_RST_*) during the calibration mode. Then, the *M_SF_* gate voltage *V_FD_* equals that of *V_PIX_SUP_* and the pixel output voltage *V_PIX_ = V_PIX_SUP_ − V_GS_* (*V_GS_* is the gate-source voltage of *M_SF_*) if ignoring the voltage drop on *M_RS_*. One has to ensure that *TG* is off to avoid disturbance into the *V_FD_* node from any charge in the PPD.

**Figure 3:**
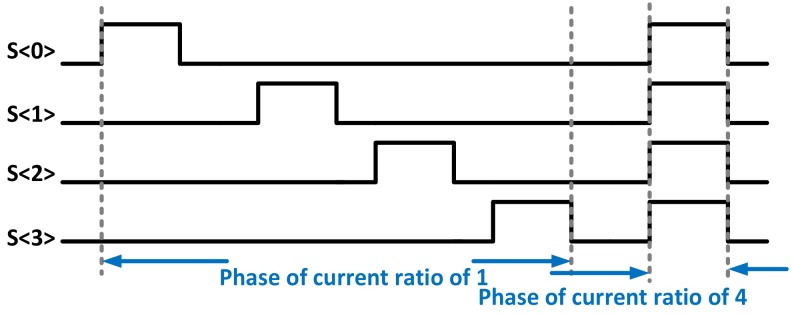
The dynamic element matching (DEM)’s timing diagram.

Therefore, the differential pixel output voltage *ΔV_PS_* at *V_PIX_*, when biased at sequential ratiometric currents Δ*I* = *I*_1_ − *I*_2_, as shown in Figure 2, is

(4) $$∆V_{PS\;}{=\;V}_{GS,2}\;-V_{GS,1}\;=\;\frac{∆I}{g_{m,SF}}$$

where *V_GS_*_,1,2_ are the gate-source voltages of the SF during the ratiometric current biasing, respectively. From Equation (4), the value of *g_m_*_,*SF*_ can be figured out through *ΔV_PS_*, as shown in Figure 2. During *T_ADC_*_,1_ and *T_ADC_*_,2_, *V_GS_*_,1_ and *V_GS_*_,2_ were quantized by the column ADC, respectively, through the pixel output *V_PIX_SUP −_ V_GS_*_,1,2_ instead of *V_GS_*_,1,2_. To enhance the calibration accuracy of *g_m_*_,*SF*_, a DEM current biasing was implemented, so that the SF can be biased with an accurate current level from 1 to 4. That is to say, the timing diagram shown in Figure 2 is simplified, as the practical calibration requires at least 15 phases to perform DEM with a ratio from 1 to 4. It was the DEM algorithm rather than the exact biasing current or voltage level that determined the accuracy of the calibration, as indicated by Equation (4). As a result, the calibrated *g_m_*_,*SF*_ depends solely on the SF itself rather than on its biasing currents. For the next step, each pixel’s current *I_1_* level will be calibrated without using the DEM. The calibration outputs from the two steps were combined together to compensate for process variations. The DEM’s timing diagram is shown in Figure 3, with a DEM ratio of 4, for illustration purposes only. The practical DEM needs at least 15 phases. As the pixel output voltage (*V_PIX_SUP_* − *V_GS_*_,1,2_) changes with its biasing current, during *I*_1_ and *I*_2_, as shown in Figure 2, the SF’s *V_TH_* changes, due to its body effect. This change of SF’s *V_TH_*, *ΔV_TH_*, was simulated to be around 10 mV at room temperature, and translates to 0.5% at an output voltage of 2 V. However, for two reasons this effect was made negligible. First of all, *V_TH_* affects the process sensor in a closed loop manner with *g_m_* and *g_mb_*, both of which are functions of *V_TH_*, as indicated in Equations (1) and (2). Secondly, both the process and the image sensors share the same SF. Therefore, the former can calibrate and compensate the latter.

### 2.3. Temperature Sensors

**Figure 4:**
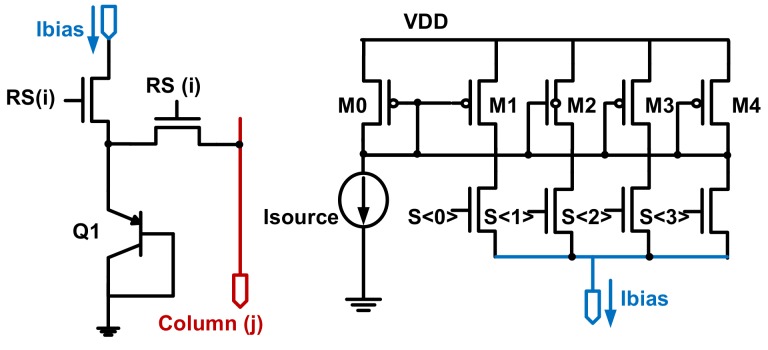
Schematic of in-pixel bipolar junction transistor (BJT)-based temperature sensor and its current biasing with DEM. M0~M4 are in practice cascode devices.

As discussed in Section 2.1, *A_SF_* has a temperature coefficient. Therefore, a few (six, in this design) temperature sensors were implemented inside the pixel array to sense the temperature locally. It was initially proposed in [9] that a single bipolar junction transistor (BJT) device, as shown in Figure 4, can serve as a test circuit on-chip and upon this principle many publications are made [3,4,10]. This structure is ideal for an incorporated temperature pixel inside a CIS array, being small sized and insensitive to device mismatch (for only a single BJT). The above advantage, however, is paid at the expense of a degraded thermal sensing coefficient at higher temperatures, due to BJT’s reverse Early effect, as mentioned in [9]. Nevertheless, this concern can be alleviated, as in this paper the temperature range of interest was until 80 °C, considering its application target in consumer electronics and its target sensing accuracy was 1 °C. As indicated in Figure 4, the differential pixel output *ΔV_BE_* at column (j), when biased at sequential ratiometric currents, is

(5) $$∆V_{BE}=\frac{kT}{q}ln(N)$$

where *N* is 4 in this design, which is the current ratio ensured by the DEM circuits shown in Figure 4. Compared to the previously published work in [3,4], the pMOS-based source follower (SF, for Q1) has been removed in this design for three purposes. First of all, Q1 or the entire cell’s output impedance was approximately 1/*g_mQ_*, where *g_mQ_* is the transconductance of Q1 and is normally at least 10 times larger than that of a MOS based SF of a similar current level. Secondly, the total temperature pixel area has been reduced to that of 1 pixel pitch, compared to 2 in [3,4]. Last but not least, a pMOS SF implemented in an n-well was acting as a parasitic photodiode and was lowering the quantum efficiency (QE) of the pixels. Specifically, a reverse biased diode (or, photodiode) existed between the n-well and the p-substrate, when the n-well was biased at a relatively high positive voltage. Furthermore, this parasitic photodiode gave rise to parasitic light sensitivity (PLS). Positive correlations between 1/PLS and QE were observed in [11]. Also, different from [3,4], in this work the BJT temperature pixels were readout by DSADCs, which are the state-of-the-art quantization circuitries for temperature sensors. Compared with the programmable gain amplifier (PGA)/CDS readout circuits employed in [3,4], the DSADC alternative in this paper has much less thermal curvature as well as noise, with the additional benefits of oversampling and noise shaping and a similar if not smaller area.

## 3. Measurement Results of SF’s Temperature and Process Dependency, Temperature, and Process Sensors

**Figure 5:**
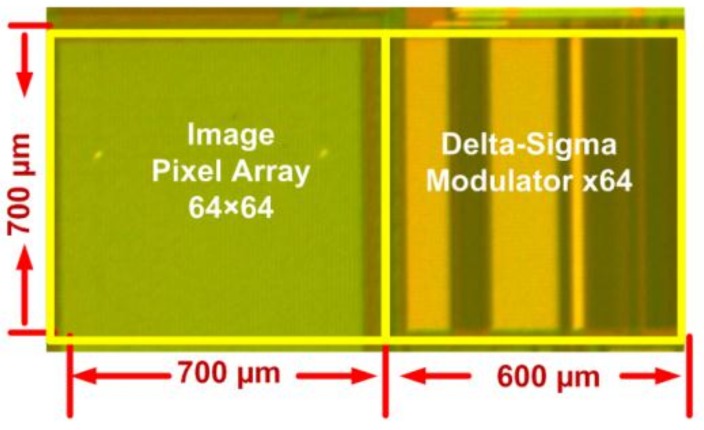
Chip micrograph of the CMOS image sensor (CIS) under test.

The measurements in this paper were performed on a 64 × 64 image pixel array prototype, as shown in Figure 5, fabricated in a 0.18 µm CIS technology. However, for reasonable results on process variability, only the center 32 × 52 pixels were used for data processing.

### 3.1. Pixel SF’s Temperature and Process Dependency

**Figure 6:**
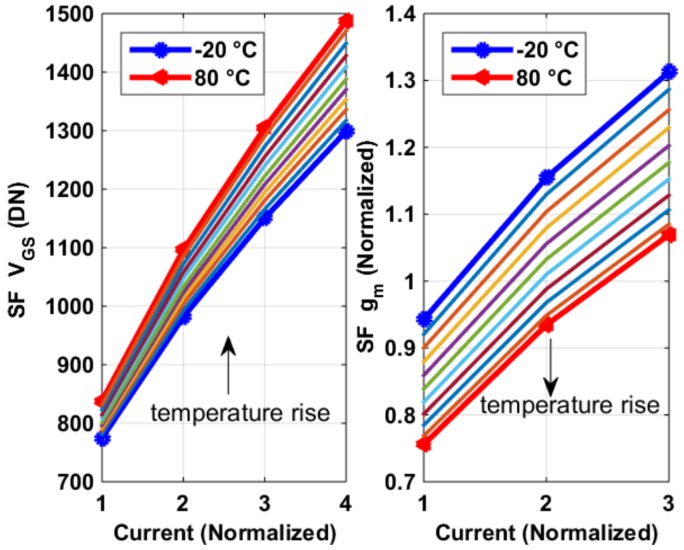
Measurement outputs of the process sensors (**left**) and their extracted *g_m,SF_*’s temperature dependency (**right**), with constant current biasing.

**Figure 7:**
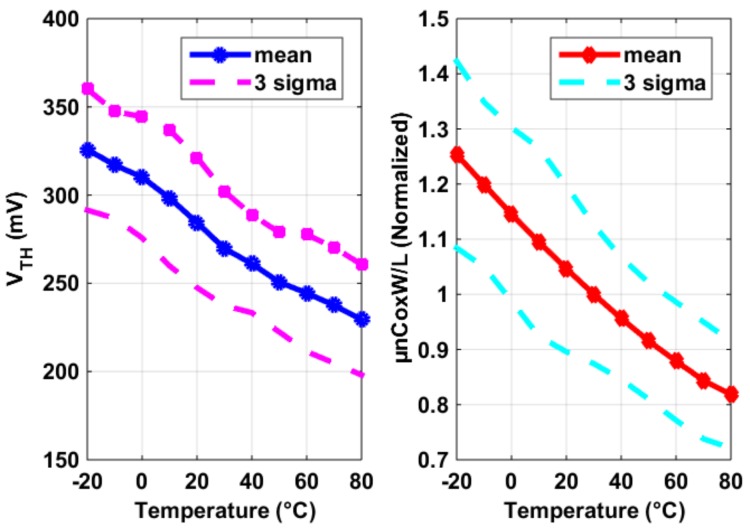
Extracted threshold voltage *V_TH_* (**left**) and *µ_n_CoxW/L* (**right**), from the measurement results of Figure 6.

**Figure 8:**
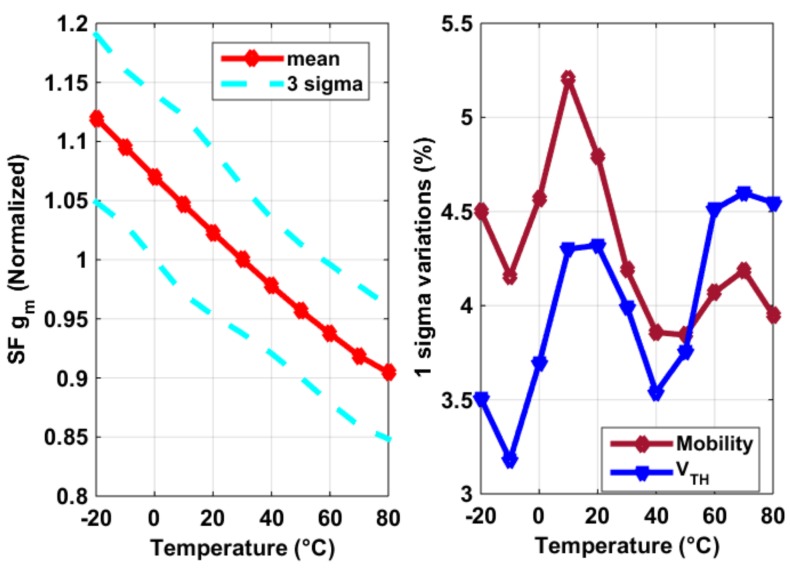
Measurement results of the SF *g_m,SF_*’s temperature dependency (**left**) and the process variability of threshold voltage *V_TH_* and *µ_n_CoxW/L* (**right**) in Figure 7, using constant current bias.

The pixel SFs’ transconductance *g_m_*_,*SF*_ were measured using the process sensors described in Section 2.2, with constant current biasing. The measurement results of average *g_m,SF_* of all pixels are shown in Figure 6, from which several observations can be made, as follows. (1) The *g_m_*_,*SF*_ decreases with temperature and increases with current biasing. (2) The *V_GS_*_,*SF*_ increases with temperature. Both observations were mainly due to degradation in surface carrier mobility *µ_n_* with temperature: (1) can be explained by *g_m_*_,*SF*_ *=* *√* (2*µ_n_C_ox_W/L·I*), (2) was caused by *V_GS_* − *V_TH_* = *√* [2*I/(µ_n_CoxW/L*)]. Here one might notice that *V_TH_* normally has a negative temperature coefficient. However, a pixel SF *V_TH_*’s temperature dependence is negligible (at least in our design), compared to that of *µ_n_*, as shown in the extraction of Figure 7, especially when the current *I* is reasonably large (which is often the case as speed is crucial for a CIS pixel). In Figure 7, the threshold voltage *V_TH_* was extracted using 2nd order best curve fitting of the I-V curve and *µ_n_CoxW/L* is exacted assuming *I* = 1/2*µ_n_CoxW/L·*(*V_GS_* − *V_TH_)*^2^, both using the results shown in Figure 6. What is also shown in Figure 7 is the 3 sigma (σ) process variability for both parameters. Figure 8 shows the thermal and process dependency of *g_m_*_,*SF*_, *V_TH_*, and *µ_n_CoxW/L*.

**Figure 9:**
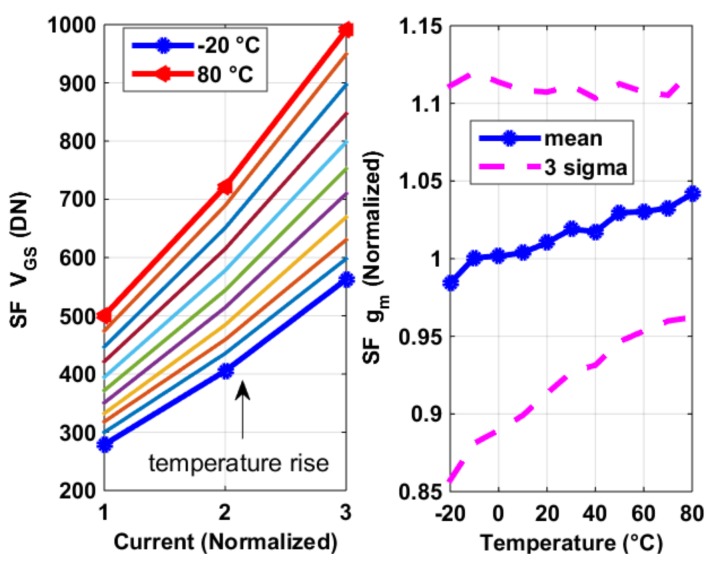
Measurement results of the SF *g_m_*_,*SF*_*’s* temperature dependency (**left**), with constant *g_m_* biasing (**right**).

**Figure 10:**
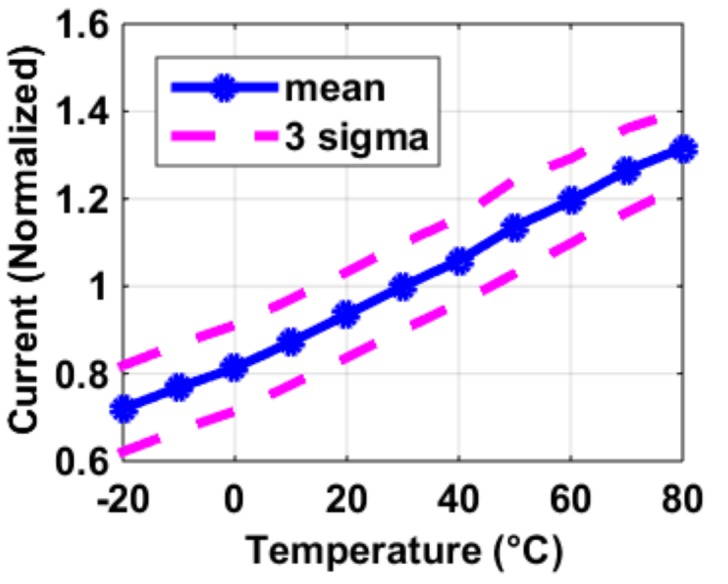
Extracted current level and temperature dependency in a constant *g_m_* biasing circuit using the measurement results of the SF’s *g_m_*_,*SF*_ in Figure 6 and Figure 9.

The SF’s transconductance g*_m_*_,*SF*_ was also measured with a different type of current biasing–constant *g_m_*, whose results are shown in Figure 9. Compared to its alternative using constant current biasing, this measurement, which has identical pixel architecture, has the following characteristics: (1) The temperature coefficient of the *g_m_*_,*SF*_ is slightly positive. This agrees with the circuit design of constant *g_m_* biasing. (2) The *V_GS_*_,*SF*_ increases with temperature. Compared to the alternative using constant current biasing, its *V_GS_*_,*SF*_’s temperature coefficient must be larger. The constant *g_m_* biasing’s actual current level and temperature coefficient were extracted based on the following assumptions: when biased with the same amount of current at the same temperature, the pixel output voltage should be the same for both measurements (constant current and constant *g_m_*), as the SFs themselves (as well as the image pixels) were of identical design and layout. Figure 10 shows the experimental extracted current which more than doubles over the temperature range of −20 to 80 °C.

### 3.2. Process Sensor and SF Voltage Gain A_SF_

**Figure 11:**
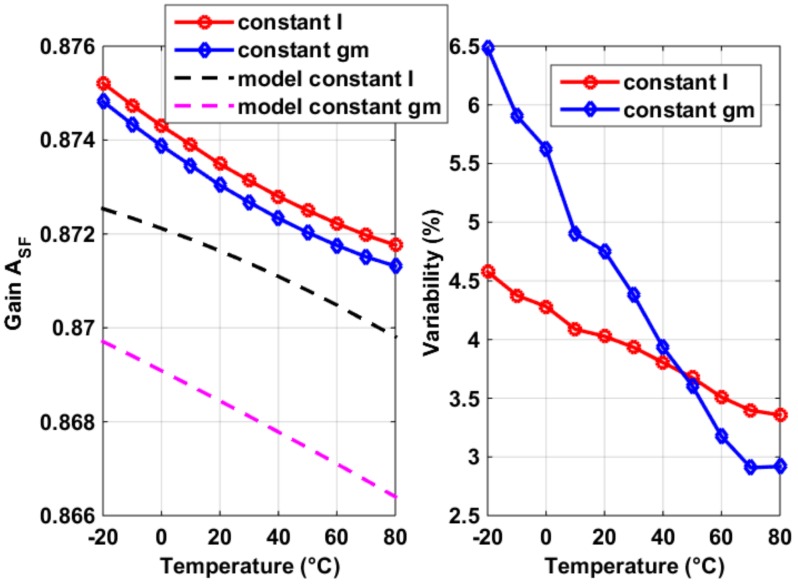
(**Left**): Measurement results of the *A_SF_* and its temperature dependency (**left**), compared with its modeled results using the process sensors’ *g_m,SF_*, for the constant current and constant *g_m_* bias circuits, respectively. (**Right**): The 1 σ process variability of measured *A_SF_*

The SF voltage gain *A_SF_* was modeled using the on-chip process sensors’ measurement results (the extracted *g_m_* as shown in Figure 6 and Figure 8), with the additional aid of the transistor-level simulated output impedance *R_L_* from Cadence, for the constant current and constant *g_m_* biasing, respectively. They were compared against the measurement results of *A_SF_*, which were obtained with decreasing *V_PIX_SUP_* voltage, as shown in Figure 11. It should be noted that the measurement of *A_SF_* was not essential to the function of the proposed process sensors, but being so enabled the verification of the process sensors’ functions. It can be seen, from the measurement results shown in Figure 11, that the proposed process sensors can model *A_SF_* as accurately as 99%.

The fact that a temperature dependency of 20% of *g_m_*_,*SF*_ in Figure 6 translates to that of 0.2% of *A_SF_* in Figure 11 is not surprising, considering the loop gain (*g_m_*_,*SF*_ + *g_mb_*_,*SF*_)·*R_L_* in Equation (1) is normally larger than 100 with cascode bias (e.g., 130 in our design at room temperature). The degradation of *A_SF_* in the closed loop is mainly from two sources. First, the decrement of *R_L_* for both biasing conditions. *R_L_* ≈ *g_m_*·*r_o_*^2^ (where *g_m_* and *r_o_* are the transconductance and output impedance of the cascode and of both transistors in the biasing, respectively). For constant *I* biasing, the degradation in *R_L_* was mainly due to that in *g_m_* (lower *µ_n_* as temperature rises) and for constant *g_m_* biasing, mostly caused by that in *r_o_* (due to increased current with temperature, as shown in Figure 10). Second, the relative increment of *g_mb_* to *g_m_*, due to decreased *V_SB_* in Equation (2), was caused by increased *V_GS_*. The process variability of *A_SF_*, as shown in the right graph of Figure 11 decreases with temperature for both biasing conditions. The explanations are the increment of (*V_GS_* − *V_TH_*) with temperature decreases mismatches in the biasing circuits as in a current source [12], for both cases. This explanation was further validated by the fact that in Figure 11 the constant *g_m_* biasing’s process variations decrease faster at higher temperatures, due to the same trend in its (*V_GS_* − *V_TH_*), compared to its constant current alternative.

### 3.3. Measurement Results of BJT Based Temperature Sensors

**Figure 12:**
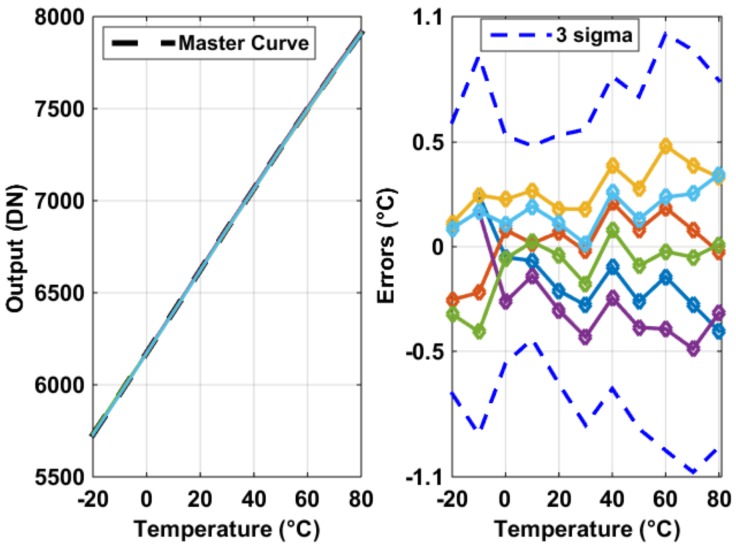
Measurement results of the six incorporated BJT-based temperature sensors inside the pixel (**left**) and their untrimmed errors, with 3 σ inaccuracies, upon a global 2nd order curve fitting (**right**).

The measurement results of the six in-pixel BJT based temperature sensors are shown in Figure 12. Each, untrimmed (uncalibrated) and upon a 2nd order master curve fitting, achieves measured inaccuracies within ±0.5 °C. The 3 σ inaccuracies of all sensors were within ±1.1 °C.

### 3.4. PGA/CDS and Constant Voltage Bias

**Figure 13:**
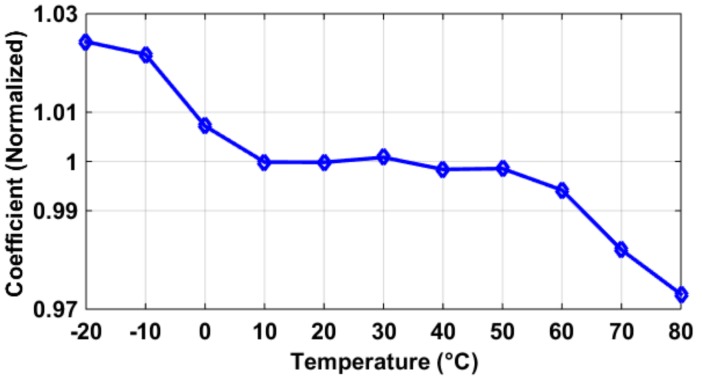
Measured temperature coefficient of a programmable gain amplifier (PGA)/ correlated double sampling (CDS) circuits implemented on the same CIS chip, with a PGA gain of 8, a settling time of 1 µs, and an unity-gain bandwidth (UBW) of 10 MHz at room temperature. This PGA/CDS circuit was not used for any other measurement in this paper.

In this design, the column readout circuit was a DSADC, which has a minimum temperature dependency. However, in many other CISs, the readout circuits, e.g., the PGA/CDS circuits may be subject to a temperature dependency as well. Nowadays, the biasing circuits are mostly constant *g_m_* ones. Therefore, for an open loop opamp, its input pair’s *g_m_* may stay almost constant, but its output impedance drops, due to the increased biasing current level to accommodate for the constant *g_m_* at higher temperature, analogous to that shown in Figure 10. The usual consequences are the opamp’s open loop gain decreases with temperature [13]. Our chip has also implemented some column-level PGA/CDS circuits, which were not employed for any measurement in this paper except in Figure 13 which shows its temperature dependency was negative. This was because the closed loop PGA gain drops as temperature increases. However, the level of any opamp’s thermal dependency is subject to its architecture (e.g., telescopic, folded cascode), design parameters, as well as how well the closed loop opamp settles within the measured time. In addition, the closed loop gain was proportional to feedback factor β, which is the 1/Gain,_PGA_, where Gain,_PGA_ is the PGA gain (e.g., 8 or 18 dB). Therefore, the larger the PGA gain, the faster the closed loop gain degrades with temperature, for identical opamp design and settling time. Meanwhile, its closed loop unity-gain bandwidth (UBW) decreases (with feedback factor β) so the settling errors within the same period increases, at the same time the closed loop gain drops, when temperature rises.

**Figure 14:**
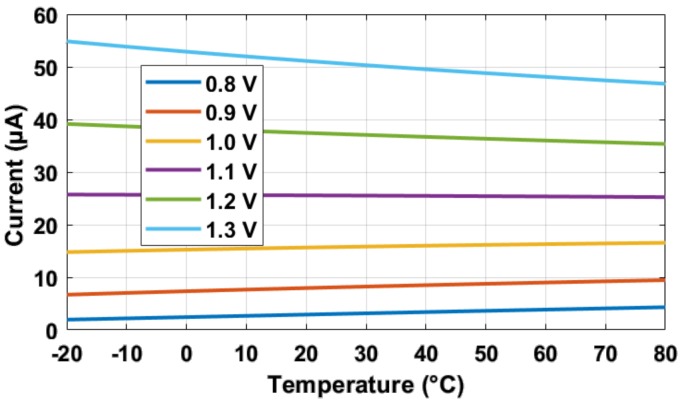
Simulation results of the current in an nMOS transistor biased with constant *V_GS_* voltage: when *V_GS_* increases from 0.8 V to 1.3 V, its current’s temperature coefficient goes from positive to negative.

Another concern was when the pixel SF was biased by a constant voltage common source transistor as in [5]. Generally speaking, as the constant voltage *V_GS_* of an nMOS transistor increases, its current’s temperature dependency changes from positive to negative [14], as shown in Figure 14. This is because *I* = 1/2*µ_n_C_ox_W/L* (*V_GS_* − *V_TH_)*^2^, where the mobility *µ_n_* and threshold *V_TH_* are against each other in their thermal effects on *I*. When *V_GS_* is small, the portion of (*V_GS_* − *V_TH_*)’s thermal influence is larger than that of *µ_n_* and vice versa. An extreme condition is that for a logic delay line, where *V_GS_* = *V_DD_*, the biasing current’s temperature coefficient is negative (with reasonably large *V_DD_*), and its propagation delay (affected by biasing/charging currents) generally increases with temperature [8]. The situations of bias currents having zero or positive temperature dependencies have been discussed in Section 3.1, and its SF gain *A_SF_* decreases slightly, to be around 0.3% over 100 °C of temperature rise, using cascode current sources.

In general, the loop gain of common source bias *g_m,SF_·r_o_* = 2/*λ*(*V_GS_*_,*SF*_ − *V_TH_*) decreases with temperature. Since *g_m_*_,*SF*_ = 2*I*/(*V_GS_*_,*SF*_ − *V_TH_*), and *r_o_* = 1/*λI* (*λ* is output impedance constant), so the open loop gain of single transistor common source bias *g_m_*_,*SF*_*·r_o_* = 2/*λ*(*V_GS,SF_* − *V_TH_*). The reason for the negative thermal dependency of loop gain is that *V_GS_*-*V_TH_* = *√* [2*I/(µ_n_CoxW/L*)] increases with temperature due to decreased *µ_n_*, invariant of the type of current source, unless the temperature coefficient of *I* is larger than that of *µ_n_*, which can rarely be the case, for the following reasons. Increased *V_GS_* level in the current source transistor raises the minimum saturation level of the pixel. In other words, a higher *V_GS_* level limits the linear dynamic range. Also, at least in our design, the fact that the thermal coefficient of *V_TH_* in the bias transistor was much larger than the pixel transistor makes it less possible for the thermal coefficient of *I* to be more negative than that of *µ_n_*. Therefore, the temperature dependence of *A_SF_* was almost always negative, despite the variations in bias circuit type. If the bias circuit is a cascode current mirror, the loop gain (*g_m,SF_* + *g_mb,SF_*)·*R_L_* ≈ *g_m_*^2^·*r_o_*^2^ is normally around 100, by which factor the thermal coefficient of *g_m_*_,*SF*_ is suppressed when it constitutes *A_SF_*, which ends up having a temperature dependency of less than 0.4% negatively. However, if the bias current circuit is a single rather than a cascode transistor, as in [5], the thermal coefficients of Equation (1) can be 10 times as large, to be around −3% or −5% over 100 °C. In addition, when there is a PGA that follows the pixel outputs, CG can degrade faster with temperature, especially with a larger PGA gain.

## 4. Measurements and Compensation of Process and Temperature Dependency in a CIS

### 4.1. Conversion Gain (CG)

**Figure 15:**
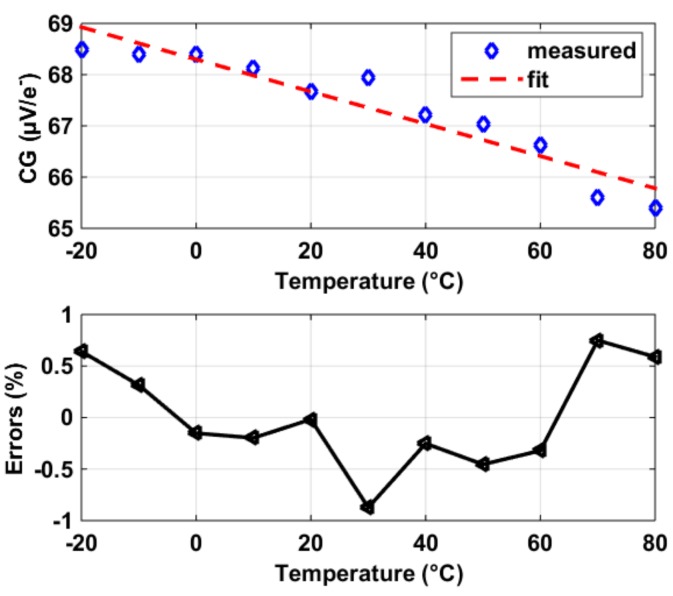
Measured conversion gain (CG) versus temperature in our CIS (**top**) and the deviations between the measurements and their fit (**bottom**).

The CG of a CIS as a function of temperature, using the constant current SF biasing, was measured and shown in Figure 15. The measured thermal dependency was around negative 5% over the measured temperature range of 100 °C. Taking into consideration that the thermal dependencies of SF were around 0.4% (last section), the 5% negative thermal coefficient of CG was mostly contributed by the positive thermal coefficient of the *C_FD_*, due to increased 1 *− A_SF_* that raised the miller capacitance associated with SF. However, various parasitic capacitors that are thermally sensitive constitute *C_FD_* [5]. In addition, the charge transfer was a transient process depending on temperature-dependent voltage levels related to Pinned Photodiode (PPD), Floating Diffusion (FD), and Transmission gate (TX) [5,6]. On the other hand, this paper specifically focuses on the thermal dependency of the SF transistor rather than all pixel voltage nodes’ thermal dependency. The reason is that this part–SF has a strong correlation with column process variability and is thermally predictable, so that can be compensated despite batch, process, or design parameter variations, compared to the rest. In this design, the thermal coefficient of CG can be modeled by an accuracy as fine as 0.5%, as shown in the bottom figure in Figure 15.

**Figure 16:**
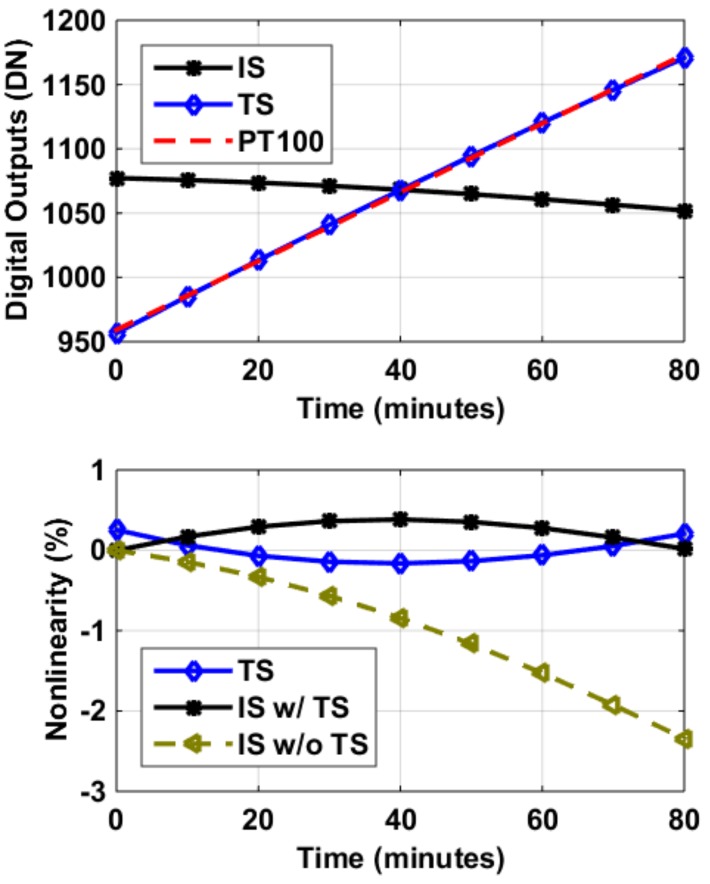
Top: Measured image sensor (IS) and temperature sensor (TS) outputs versus time, along with a reference temperature sensor (PT100). Bottom: when the temperature changes from 20 to 60 °C gradually in a temperature chamber, nonlinearity caused by the thermal drift (IS w/o TS) was corrected with the temperature information provided by the on-chip TS (IS w/TS). The correction was done digitally and on-the-fly.

To test the design’s capability to compensate for dynamic temperature change, both the temperature and the image pixels were measured at the same time. Figure 16 shows the measurement results of the aforementioned experiments. Measurements were done while all sensors were heated up from 20 to 60 °C in a temperature chamber, gradually. The image sensors’ outputs drop with time, caused by temperature drift, giving rise to more than 2% of non-linearity, which has been compensated using the thermal information provided by the temperature sensors whose outputs increase with time.

Among the 2.3% of thermal induced nonlinearity, 2% was corrected, to be less than 0.3% eventually. That was an 87% improvement, compared to the case without using the on-chip temperature sensors for dynamic thermal compensation.

### 4.2. Dark Current and DSNU

**Figure 17:**
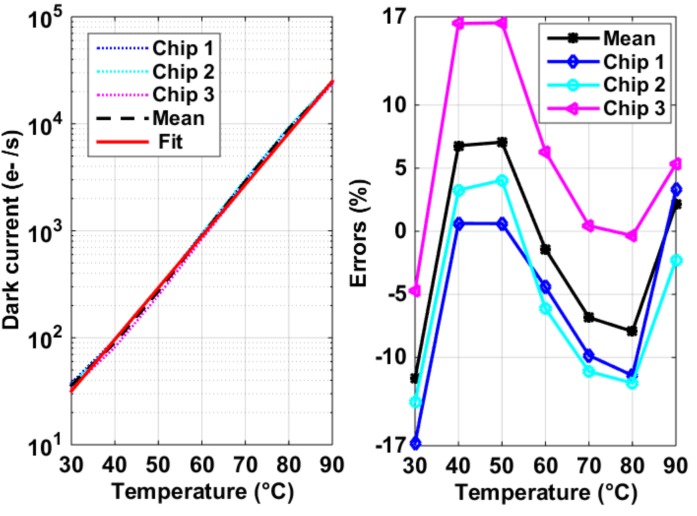
Measured average dark current for three chips and their exponential fit (**left**) and deviations from the exponential fit (**right**).

On one hand, the DSNU in a 4T PPD pixel is caused by variations among dark currents from pixel to pixel [1]. On the other, the average dark current dependency on the temperature of a CIS array fabricated using the same pixel architecture and readout circuits can be predicted by an exponential fit (*y = a·exp(b·T*)), where *T* refers to the temperature and *a*, *b* are constants. Figure 17 shows the measured average dark current from three chips and their global exponential fit. It also shows the derivations between the measurements and their fit were within in ±17% for three chips. In this way, the average dark current can be predicted and compensated with an accuracy of at least 83%. The average dark current was measured to be around 30 e^−^/s at room temperature and doubled almost for every 6 °C of temperature rise.

**Figure 18:**
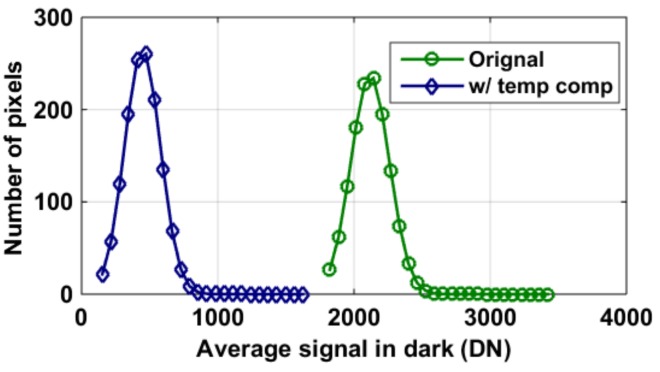
Histogram of measured dark signal and dark signal non-uniformity (DSNU). Original: measured at 60 °C and 250 ms, averaged 100 frames; w/ temp comp: compensation by subtracting the reference dark current at room temperature, along with predicted dark current using temperature information (as shown in Figure 17).

Figure 18 shows the dark signal histogram when a dark frame was taken at 60 °C and 250 ms. Originally, the average dark signal and DSNU were 2118 DN and 141 DNrms. Upon cancelling the image offset with a reference image taken at room temperature, the DSNU was reduced by 10 DNrms to 131 DNrms. With the additional aid of the average dark signal’s temperature fit shown in Figure 17, dark signal was reduced by 79% to 446 DN. The method of “w/temp comp”, facilitated with in-pixel temperature sensors and with an additional aid of a dark frame captured at room temperature, eliminated the need to capture a dark frame before each image, thus improving the readout speed and getting rid of a physical shutter.

## 5. Conclusions

This research paper analyzes and compensates for the process and the temperature dependency in a CIS image sensor, facilitated with the temperature and the process sensors implemented inside the image pixel array. Compared to previous publications, the new features of this paper are as follows. (1) The proposed process sensors were based on measuring the imager pixel’s SF *g_m_*_,*SF*_ and were verified against the measurement results of *A_SF_*, in a 32 × 52 pixel array. (2) The process and thermal variations of *A_SF_* were measured while those of *V_TH_*, *g_m_*_,*SF*_, and *A_SF_* were extracted using measurement results. Especially, the sources contributing to thermal dependence of *A_SF_* have been analyzed, for various cases, from constant current and constant *g_m_* biasing current sources, to the general situation of a constant voltage bias. The conclusions are that if one can afford a cascode current biasing, *A_SF_*’s temperature dependency would be less than 0.5% over a temperature range of 100 °C, due to the loop gain around 100 that biases the SF. Otherwise, if a single transistor is employed as a current source, *A_SF_*’s temperature dependency can be ten times as large, to be negative 5% over the same temperature range. (3) The thermal dependency of CG was measured to drop around 5% over 100 °C of temperature change, mainly attributed to that of *C_FD_* rather than that of *A_SF_*. (4) The proposed incorporated BJT-based temperature sensor occupies an area of 11 × 11 µm, and provides an untrimmed accuracy better than ±0.5 °C over the temperature range between −20 and 80 °C. Compared to previous publications on temperature sensors as listed in Table 1, the advantages of our temperature sensors are a much smaller area, better untrimmed accuracy, and reasonable figure of merit (FOM) [17]. Using the temperature information provided by the temperature sensor, the non-linearity of the CIS outputs caused by thermal drift of CG can be corrected by more than 87%. The average dark current can be predicted by at least 83% and dark signal can be compensated by at least 79%, respectively.

**Figure 19:**
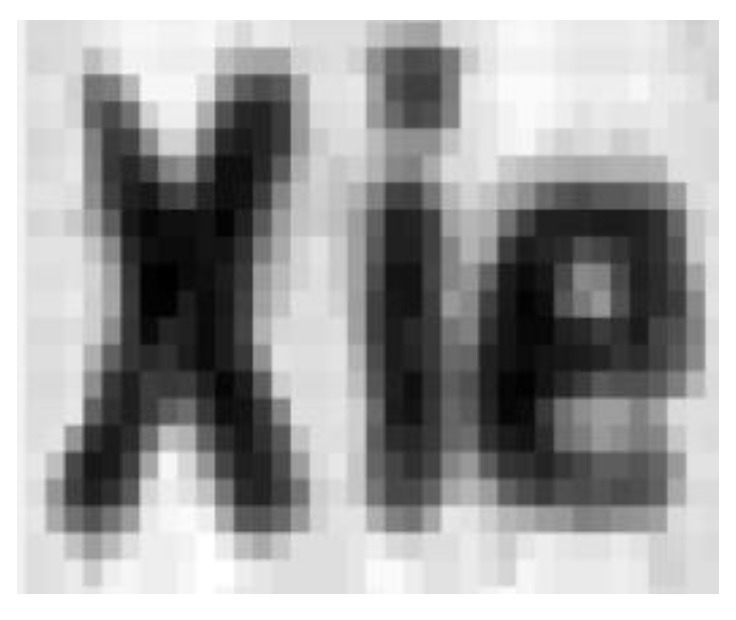
An image taken by part of the CIS (64 × 27), with constant current bias.

In summary, the measurement results obtained, and the methods proposed in this paper may serve as guidelines, rather than the ultimate solutions to compensate for thermal and process dependencies for CIS. Figure 19 shows an image taken by our low-resolution prototype CIS. In the future, a larger size array is planned to be measured for better understanding of process variability in practical image sensors.

## Figures and Tables

**Table 1:** Summary of performances

	This Work	[15]	[16]
Sensor Type	BJT	BJT	MOS
CMOS Technology	0.18 µm	0.13 µm	28 nm
Area (µm^2^)	121	60,000	1000
Temperature Range	−20 °C to 80 °C	−20 °C to 100 °C	−5 °C to 85 °C
3 σ accuracy	±1.1 °C	−1.7/1.26 °C	−3.3/1.9 °C
Calibration	Un-trimmed	Two-point	One-point
Power Consumption (µW)	36	744	56
Conversion Time (ms)	16	13.3	0.036
Resolution (°C)	0.09	0.187	0.76
Resolution FOM (nJ∙K^2^)^a^	4.6	346	1.2
Rel.IA (%)^b^	2.2	2.4	5.8

^a^ Energy/Conversion × (Resolution)^2^, in reference to [17], ^b^ 3 σ accuracy/temperature range, in reference to [17].
